# Case report: Conservative treatment for fertility preservation in a woman with hemoperitoneum due to an invasive mole

**DOI:** 10.3389/fonc.2022.1019082

**Published:** 2022-12-06

**Authors:** Linjuan Xu, Min Feng, Jing Cai, Hongmei Li

**Affiliations:** ^1^ Department of Obstetrics and Gynecology, Union Hospital, Tongji Medical College, Huazhong University of Science and Technology, Wuhan, China; ^2^ Department of Gynecology, Children’s Hospital of Shanxi and Women Health Center of Shanxi, Taiyuan, China

**Keywords:** invasive mole, hemoperitoneum, conservative treatment, case report, uterine rupture

## Abstract

**Background:**

Invasive moles are a subtype of gestational trophoblastic neoplasia (GTN) that usually develops after hydatidiform molar pregnancies. Uterine rupture in high-risk GTN is a rare and potentially catastrophic event. The treatment of invasive mole perforation with uterine rupture is particularly challenging in young women who desire fertility preservation.

**Case presentation:**

We present the case of a 22-year-old woman with a rapidly transformed invasive mole after two evacuations for a complete molar pregnancy. Within 21 days of the second molar evacuation, the serum β-hCG level surged from 5,718 mIU/ml to 444,617 mIU/ml. An ultrasonography examination showed the uterus was 9.2×8.9×7.8 cm in size with an uneven echo area of 6.9×5.2 cm near the fundus of the uterine cavity; the convex anterior wall had no normal muscle layer, and the outer margin was about 0.24 cm from the serosal layer. The patient was diagnosed with an invasive mole. Since she desired fertility preservation, we proposed a methotrexate (MTX) chemotherapy regimen. Before the planned chemotherapy, she experienced sudden abdominal pain accompanied by a blood pressure of 76/48 mmHg and a pulse rate of 116 bpm. An emergency abdominal ultrasound scan showed acute intra-abdominal bleeding (approximately 2,000 ml), and blood tests showed a hemoglobin concentration of 7.9 g/dL. Immediate uterine artery embolization was performed, and 35 mg MTX was administered bilaterally through the uterine arteries. The next day, the serum β-hCG decreased to 83,530 mIU/ml, and the vital signs remained stable. Seven days later, the patient received a combination of etoposide, methotrexate, dactinomycin, cyclophosphamide, and vincristine (EMACO), and the serum β-hCG level normalized after cycle five. At the 13-month follow-up after therapy completion, the woman was disease-free with a normal β-hCG level.

**Conclusion:**

Our experience highlights the potential feasibility and efficacy of conservative treatment for fertility preservation in such scenarios.

## Introduction

Invasive hydatidiform mole is pathologically classified as a borderline or biologically uncertain tumor by the WHO (1) and is clinically classified as a malignant tumor combined with choriocarcinoma as gestational trophoblastic neoplasia (GTN). Owing to its unique histological origin and biological behavior, GTN is the first solid tumor that can be cured by chemotherapy (2–4).

Although most invasive moles can be cured with chemotherapy, hysterectomy is often performed if the uterus ruptures and is life-threatening (5–7). However, patients that experience this condition are usually young adults. If they desire fertility preservation, fertility-sparing surgery should be performed, such as resection of the tumor with uterine reconstruction and bilateral uterine artery embolization using gelfoam slurry to temporize bleeding before surgery (8–10). Nonetheless, conservative treatment has not yet been reported in the literature.

Here, we report the case of a young woman with acute uterine rupture and hemorrhagic shock caused by an invasive mole. Conservative fertility-sparing intervention was performed, and a favorable prognosis was achieved.

## Case description

**Figure 1:**
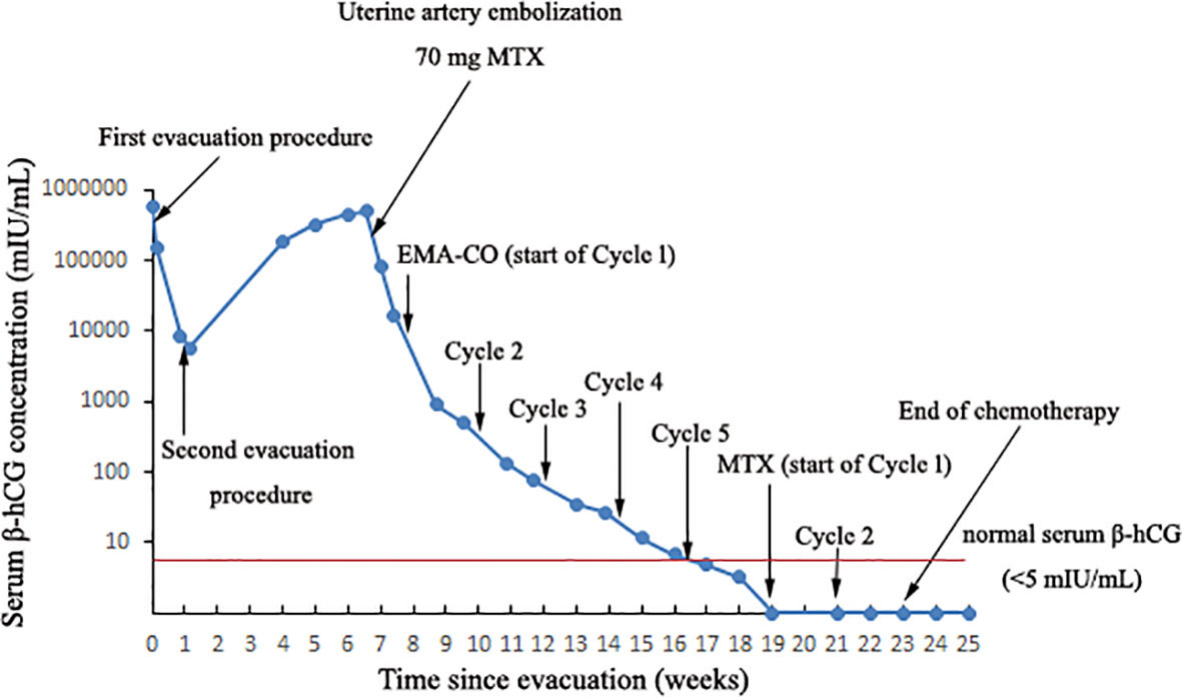
The curve showing serum β-hCG during the invasive mole development and in response to the treatment procedures.

**Figure 2:**
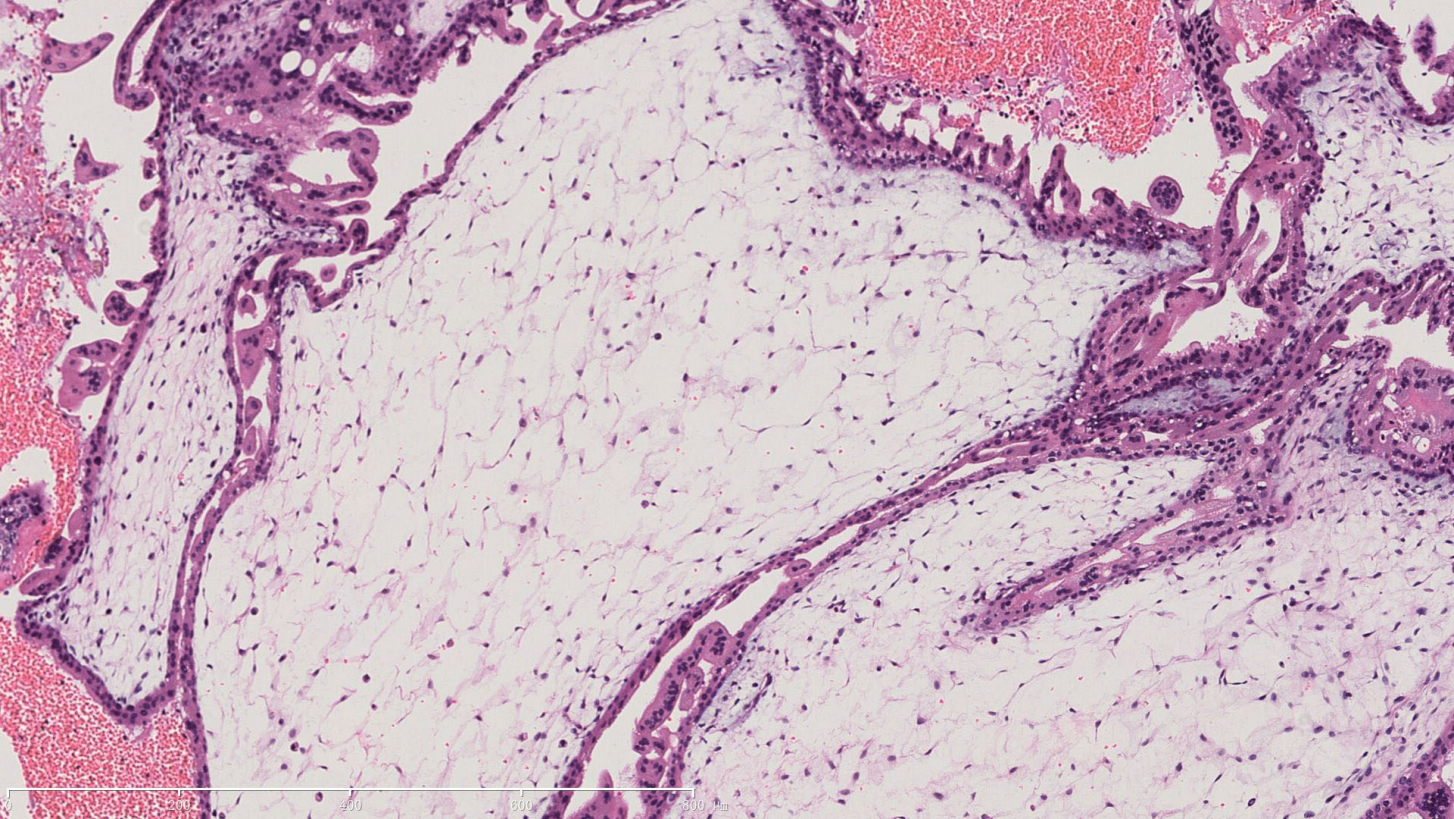
Pathological characteristic of the invasive mole. A representive image of HE staining demonstrating villi edema and trophoblast cells proliferation.

A 22-year-old woman (married, gravida 0, para 0) presented to our department with cessation of menstruation for 73 days and slight vaginal bleeding for 4 days. She reported no nausea, vomiting, abdominal pain, or bloating. Physical examination revealed a small amount of blood in the vagina and an abnormally enlarged, soft uterus. The serum β-hCG was 582,621 mIU/ml. Transabdominal ultrasound showed the uterus was 10.4 × 11.0 × 7.7 cm, with an uneven echo area of 8.7 × 6.6 cm in the uterine cavity, which looked like falling snow. The patient underwent suction and curettage, and a pathological examination revealed a complete hydatidiform mole. A week later, an ultrasound examination suggested a residual lesion in the uterine cavity (2 × 1.5 cm), and the β-hCG was 8,407 mIU/ml. Subsequently, she underwent a second suction and curettage, and the β-hCG level further decreased to 5,718 mIU/ml (**Figure 1**). The results of the second pathological examination were consistent with those of the first (**Figure 2**).

**Figure 3:**
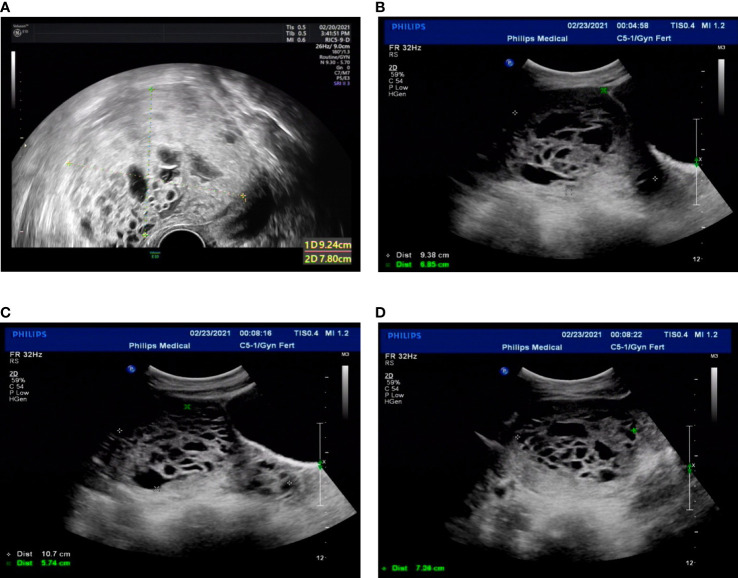
Transvaginal ultrasound. **(A)** Twenty-one days after the second molar evacuation. **(B-D)** The ultrasonographic image when the uterus perforation. The uterus was 9.4 × 9.7 × 6.9 cm, an uneven echo area of 10.7 × 7.1 × 5.7 cm was observed in uterine cavity and cervical canal, protruding left anterior wall, local muscle layer was unclear.

**Figure 4:**
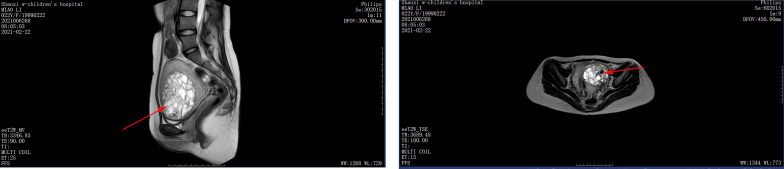
Pelvic MRI. Twenty-one days after the second molar evacuation, pelvic MRI scan showing a 7.1 × 6.1 × 8.2 cm mass invading the left anterior uterine wall, extending from the muscular layer to the serosal layer.

The patient was then recommended weekly β-hCG assessments for surveillance. However, she did not comply with the follow-up schedule. Within 21 days after the second molar evacuation, her serum β-hCG level was elevated to 183,309 mIU/ml, despite an unremarkable ultrasonography examination. She didn’t take the high hCG level seriously so that she forgot to consult doctor in time. After that, the β-hCG level continued to rise, reaching 489,190 mIU/ml on day 39 after the second procedure. An ultrasonography examination showed that the uterus was 9.2 × 8.9 × 7.8 cm in size, an uneven echo area of 6.9 × 5.2 cm was near the fundus of the uterine cavity, the convex anterior wall had lost its normal muscle layer, and the outer margin was about 0.24 cm from the serosal layer (**Figure 3A**). Pelvic magnetic resonance imaging (MRI) showed an enlarged uterus with multiple cystic mixed abnormal signals in the left anterior wall. The lesion was about 7.1 × 6.1 × 8.2 cm, extending from the muscular layer to the serosal layer (**Figure 4**). The patient was diagnosed with an invasive mole. Chest and cranial CT scans were performed, and no metastatic lesions were detected. These findings indicated an invasive mole at stage I according to the FIGO 2000, with a World Health Organization (WHO) score of 6, and belonged to the low-risk GTN.

The patient was very young and had the willing to perserve her fertility, and we proposed methotrexate (MTX) chemotherapy. The day before the planned chemotherapy, she experienced sudden abdominal pain accompanied by shortness of breath, blood pressure of 76/48 mmHg, and a pulse rate of 116 bpm. An emergency abdominal ultrasound scan showed acute intra-abdominal bleeding (approximately 2,000 ml) (**Figures 3B–D**), and blood tests showed a hemoglobin concentration of 7.9 g/dL. We recommended emergency surgery, but she rejected. Given she was experiencing an acute intra-abdominal bleeding which risked her life, then we chose to perform the MTX chemical therapy and uterine artery embolization simultaneously which can suppress the tumor and stop bleeding at the same time, and is a relative conventional way to stop bleeding in a short time. So uterine artery embolization was performed, and 35 mg of MTX was administered bilaterally through the uterine arteries. Simultaneously, four units of packed red blood cells (RBCs) were transfused. The next day, the serum β-hCG decreased to 83,530 mIU/ml, and the hemoglobin concentration was 8.2 g/dL. Four units of packed RBCs were transfused again. There were no signs of persistent intraperitoneal bleeding, and the patient was stable. Seven days later, chemotherapy with etoposide, methotrexate, dactinomycin, cyclophosphamide, and vincristine (EMACO) was administered. After the first cycle, the serum β-hCG dropped to 894.2 mIU/ml and was normalized after cycle five. Consider the cost and the severe side effects such as myelosuppression, vomiting, hair loss and so on. We decided to use the MTX regimen with 1mg/kg/day intramuscularly on days 1, 3, 5, 7, with folinic acid 0.1 mg/kg/day orally 24 hours after MTX on days 2,4,6,8); repeat every 2 weeks. The patient received two additional courses of chemotherapy with MTX to reduce the risk of relapse. At the 13-month follow-up after therapy completion, the patient was disease free with negative β-hCG.

## Timeline

### Diagnostic assessment

Invasive mole is a form of gestational trophoblastic neoplasia (GTN) that most commonly occurs after the evacuation of a molar pregnancy. Most cases are clinically rather than histologically diagnosed.

- Diagnostic methods: The diagnostic criteria for GTN after hydatidiform mole were as follows (1): The elevated blood β-hCG level reached a plateau ( ± 10%) for 4 times (day 1, 7, 14, 21), lasting for 3 weeks or longer (2); The serum β-hCG level increased (>10%) for 3 consecutive times (day 1, 7, 14) for 2 weeks or longer (3); The histological diagnosis was invasive hydatidiform mole.
- Diagnostic challenges: It is necessary to exclude residual pregnancy (any retained molar tissue/products of conception after suction and curettage) and repeat pregnancy during diagnosis. If it cannot be excluded, it is recommended to repeat suction and curettage, and if necessary, hysteroscopy can be performed.
- Diagnosis (including other diagnoses considered): The patient was diagnosed with an invasive mole. Chest and cranial CT scans were performed, and no metastatic lesions were detected. These findings indicated an invasive mole at stage I according to the FIGO 2000, with a World Health Organization (WHO) score of 6.
- Prognostic characteristics when applicable: β-hCG is a reliable indicator of tumor activity. At the 13-month follow-up after therapy completion, the patient was disease free with negative β-hCG.

## Discussion

Invasive mole typically develops after evacuation of a molar pregnancy. The interval from an antecedent molar pregnancy is usually less than six months, and the earliest is one week (11). In this case report, the patient had a history of complete hydatidiform mole within 21 days after curettage. Standard follow-up examinations are very important to detect and diagnose invasive mole in time. If this patient had perfectly adhered to the weekly follow-up schedule, the uterine perforation and hemorrhagic shock might have been avoidable.

Invasive moles are highly sensitive to chemotherapies. Since the late 1950s, it has been proven that high-dose MTX could effectively cure tumors. With low dose methotrexate, complete remission is achieved in most non metastatic and low-risk cases (12). Then a series of effective chemotherapy drugs have been discovered. Therefore, chemotherapy is preferred for the treatment of invasive moles (13). The best treatment for drug-resistant lesions or lesions with perforation and bleeding has not been established; these patients are most often managed with highly individualized multimodality therapy, incorporating chemotherapy, with surgery, arterial embolization, or localized radiation therapy (14).

Uterine rupture in high-risk GTN is a rare and potentially catastrophic event (5, 8, 9, 15). The Risk factors for uterine rupture include previous surgery involving the myometrium, trauma, abnormal uterine development and thin local uterine muscle layer (16). Our patient underwent suction and curettage twice, which might have caused damage to the uterine wall and contributed to the rupture. To our knowledge, all previously reported cases of invasive mole perforation with active bleeding were managed with surgery (5–10). Further, some women with uterine perforation were managed with hysterectomy, and fertility preservation was not considered (5–7). Some reports support the feasibility of fertility-preserving surgery in women who experience life-threatening hemorrhage caused by uterine rupture (8–10). Zamani et al. (2021) reported a 21-year-old woman with hemoperitoneum due to an invasive mole who underwent uterine lesion resection and repair of the uterine wall (8). David-West et al. (2020) described a case of high-risk GTN complicated by uterine rupture. She was treated with bilateral uterine artery embolization followed by exploratory laparotomy to control the hemorrhage (9). Among the 78 patients with GTN who achieved fertility-sparing uterine lesion resection reported in the study performed by Wang et al. (2017), only five patients underwent surgery due to suspected uterine rupture (10). All five patients were treated with bilateral uterine artery embolization to terminate excessive vaginal bleeding before fertility-sparing uterine lesion resection. However, conservative treatment has not yet been reported in the literature. For the first time, we report the case of a young woman with acute uterine rupture and hemorrhagic shock caused by an invasive mole; conservative fertility-sparing intervention with uterine artery embolization combined with chemotherapy was performed, and a favorable prognosis was achieved. Therefore, regarding uterine rupture with an invasive mole, if the bleeding occurs during hospitalization or the real-time hemoglobin concentration is greater than 7.0 g/dL, uterine artery embolization followed by chemotherapy may be a feasible and safe conservative treatment.

In conclusion, invasive moles can develop and progress after a complete molar pregnancy. Standard follow-up examinations are important for detecting and diagnosing invasive moles. In cases of uterine rupture in young patients with a desire for fertility preservation, uterine artery embolization can be used to control the acute phase, followed by chemotherapy, effectively controlling the disease in time without the need for surgical treatment of the uterus.

## Data availability statement

The original contributions presented in the study are included in the article/supplementary material. Further inquiries can be directed to the corresponding author.

## Ethics statement

All procedures performed in studies involving human participants were in accordance with the ethical standards of the institutional and/or national research committee and with the 1964 Helsinki declaration and its later amendments or comparable ethical standards. Informed consent was obtained from the patient described in this case report.

## Author contributions

Conceptualization, LX and HL; investigation, LX, JC, and MF; data curation, LX and MF; writing—original draft preparation, LX; writing—review and editing, LX, MF, JC, and HL. All authors have read and agreed to the published version of the manuscript.
